# Supplementary material for: Identifying the most effective essential medicines policies for quality use of medicines: A replicability study using three World Health Organisation data-sets
Source: PLoS One. 2020 Feb 6;15(2):e0228201. doi: 10.1371/journal.pone.0228201 (PMC7004360; doi:10.1371/journal.pone.0228201)
Supplement: S4 Table — (DOCX) [file pone.0228201.s004.docx]

Supporting Information Table S4:

Linear regression analyses* of individual QUM indicators versus number of effective policies (out of 18) countries reported implementing

| QUM indicator | Beta coefficient ( β ) | Correlation coefficient ( r ) | Degrees of freedom | p-value |
| --- | --- | --- | --- | --- |
| % patients prescribed antibiotics | - 0.84 | - 0.37 | 1, 35 | < 0.05 |
| % patients not needing antibiotics that were prescribed them | - 1.02 | - 0.17 | 1, 12 | > 0.05 |
| % upper respiratory tract infection cases treated with antibiotics | - 2.10 | - 0.56 | 1, 19 | < 0.05 |
| % pneumonia cases treated with an appropriate antibiotic | + 0.77 | + 0.17 | 1, 16 | > 0.05 |
| % diarrhoea cases treated with antibiotics | - 1.45 | - 0.24 | 1, 15 | > 0.05 |
| % diarrhoea cases treated with oral rehydration solution | + 1.68 | + 0.37 | 1, 15 | > 0.05 |
| % diarrhoea cases treated with anti-diarrhoeal drugs | - 3.29 | - 0.49 | 1, 7 | > 0.05 |
| % malaria cases treated with an appropriate anti-malarial | + 2.14 | + 0.35 | 1, 17 | > 0.05 |
| % prescribed drugs belonging to the Essential Medicines List | + 0.92 | + 0.33 | 1, 26 | > 0.05 |
| % drugs prescribed by generic name | + 1.44 | + 0.26 | 1, 30 | > 0.05 |
| % patients prescribed vitamins (mainly B complex & multivitamin) | - 0.81 | - 0.28 | 1, 12 | > 0.05 |
| % patients prescribed injections | - 0.96 | - 0.28 | 1, 29 | > 0.05 |
| % patients treated in compliance with standard treatment guidelines | + 0.60; (+ 1.45)** | + 0.1; (0.28)** | 1, 18; (1, 11)** | > 0.05; (>0.05)** |

* QUM indicator = constant + β (Number of policies out of 18 reported implemented) + error

** Sensitivity analysis which included only those country surveys which measured STG compliance in more than two diseases, i.e. which excluded vertical disease programs covering one illness and in which the process of judging STG compliance varied widely.
